# Sudden emergence of human infections with H7N9 avian influenza A virus in Hubei province, central China

**DOI:** 10.1038/s41598-018-20988-9

**Published:** 2018-02-06

**Authors:** Jiafa Liu, Junqiang Xu, Linlin Liu, Xiaoman Wei, Yi Song, Bin Fang, Xiao Yu, Xiang Li, Guojun Ye, Yingying Du, Mingyue Chen, Weifeng Shi, Di Liu, Edward C. Holmes, Jie Cui

**Affiliations:** 1 Hubei Provincial Center for Disease Control and Prevention, Wuhan, 430079 China; 2 CAS Key Laboratory of Special Pathogens and Biosafety, Center for Emerging Infectious Diseases, Wuhan Institute of Virology, Chinese Academy of Sciences, Wuhan, 430071 China; 3 University of Chinese Academy of Sciences, Beijing, 100049 China; 4 Institute of Pathogen Biology, Taishan Medical College, Taian, Shandong 271000 China; 5 Center for Influenza Research and Early-Warning (CASCIRE), Chinese Academy of Sciences, Beijing, 100101 China; 6 CAS Key Laboratory of Pathogenic Microbiology and Immunology, Institute of Microbiology, Chinese Academy of Sciences, Beijing, 100101 China; 7 Savid Medical School, University of Chinese Academy of Sciences, Beijing, 101408 China; 8 Marie Bashir Institute for Infectious Diseases and Biosecurity, Charles Perkins Centre, School of Life and Environmental Sciences and Sydney Medical School, The University of Sydney, Sydney, New South Wales Australia

## Abstract

There have been five waves of H7N9 avian influenza virus (AIV) infection in humans since its initial emergence in China in 2013, posing a significant threat to public health. Hubei province was free local transmission during the first four waves of H7N9 AIV. However, multiple cases of human H7N9 infection were reported in Hubei during January 2017. To understand the molecular epidemiology that underlies this sudden emergence, we collected samples from 14 human cases of H7N9 influenza virus from Hubei province, along with environmental samples from different locations in Hubei. Our analysis revealed that the newly emerged human H7N9 viruses were all from persons exposed to poultry and shared the same origin as the environmental sampled viruses in the Yangtze River lineage of H7N9. Notably, we also documented an earlier and distinct importation from Jiangsu province that may have established a local environmental reservoir. Our study highlights the need for continued surveillance of H7N9 in both human and avian populations in central China.

## Introduction

Avian influenza viruses (AIVs) pose an important and continuous threat to public health. Since the first human infection with H7N9 AIV was identified in March 2013^1^, there have been five outbreak waves in China during 2013–2017. As of 28th June 2017, there have been 1568 confirmed cases of human H7N9 infection and 599 deaths (http://www.fao.org/ag/againfo/programmes/en/empres/H7N9/situation_update.html). With the exception of the first wave (February 2013–September 2013), all H7N9 AIV outbreaks have occurred during the winter–spring seasons, beginning in October, with cases increasing in number in late December, and peaking in January of the following year^2,3^. However, in the fifth wave the epidemic began one month earlier, with a sharp increase in human cases at the start of 2017^3,4^. Importantly, human infection with highly pathogenic H7N9 AIV in Guangdong province, China, was also reported during the fifth wave, with an insertion of multiple basic amino acids at the HA cleavage site associated with enhanced virulence^5–7^.

According to previous phylogenetic analyses, two lineages of H7N9 have been established in China, the Yangtze River Delta lineage (Zhejiang, Jiangsu, Anhui provinces and Shanghai municipality) and the Pearl River Delta lineage (Guangdong province, Hong Kong and Macao special administrative region), with the former widely distributed and the original source of the H7N9 outbreaks in humans^8^. No avian, human, nor environmental infections with H7N9 AIVs were documented in the first four waves in Hubei province despite annual surveillance performed by the Hubei Provincial Center for Disease Control and Prevention (CDC), with the exception of two imported infections in 2015 and 2016. However, since January 2017 several H7N9 strains have been isolated from both human (n = 12) and environmental samples in Hubei province by the Hubei CDC. We performed phylogenetic analyses on these human and environmental samples to determine their origins and evolution.

## Materials and Methods

### H7N9 AIVs data collection

Human samples (n = 12) were collected from the respiratory tract of the suspected H7N9 infection in Hubei province, China, during January 2017–Febuary 2017. To these samples we added A/Hubei1/2016, an imported case in which the patient was infected in Jiangsu province in 2016, and A/Hubei/34007/2015 which was downloaded from GISAID (platform.gisaid.org), with the patient infected by chickens imported from Anhui province during 2015. All patients (n = 14, Table 1) with H7N9 infection experienced severe pneumonia.

**Table 1:** H7N9 viruses sampled from humans in Hubei province, China.

Isolate name	Collection date (YY-MM-DD)	Passage history	Gender	Age (years)	Occupation	Live poultry-related exposure	Infection area	Outcome
A/Hubei/09906/2017	2017-01-24	E1^b^	Female	78	Farmer	LPM^c^	Xiaogan	Died
A/Hubei/09907/2017	2017-01-31	E1	Male	55	Construction worker	LPM	Xiaogan	Died
A/Hubei/09909/2017	2017-02-07	E1	Female	26	Unknown	LPM	Ezhou	Unknown^e^
A/Hubei/09910/2017	2017-02-03	E1	Male	35	Garageman	Backyard poultry^d^	Wuhan	Unknown
A/Hubei/09911/2017	2017-02-04	E1	Male	28	Food vendor	LPM	Xianning	Unknown
A/Hubei/09912/2017	2017-02-04	E1	Male	29	Garment factory worker	LPM	Wuhan	Unknown
A/Hubei/09913/2017	2017-02-06	E1	Male	74	Retiree	Backyard poultry	Wuhan	Died
A/Hubei/09914/2017	2017-02-06	E1	Male	72	Retiree	LPM	Wuhan	Died
A/Hubei/09929/2017	2017-01-26	E1	Male	46	Construction worker	Backyard poultry	Wuhan	Unknown
A/Hubei/09937/2017	2017-02-04	E1	Female	71	Farmer	Backyard poultry	Huanggang	Unknown
A/Hubei/1/2016	2016-03-13	E2	Female	43	Poultry worker	LPM	Nanjing	Unknown
A/Hubei/11944/2017	2017-02-20	E1	Female	74	Retiree	LPM	Xianning	Unknown
A/Hubei/11950/2017	2017-02-20	E1	Male	40	Farmer	LPM	Xiaogan	Unknown
A/Hubei/34007/2015^a^	2015-04-22	E1	Male	50	Poultry worker	LPM	Huanggang	Unknown

^a^The genome sequence of A/Hubei/34007/2015 was downloaded from GISAID.

^b^E1 refers to the fact that this isolate was passaged once in embryonated hens’ eggs.

^c^LPM refers to persons exposed to live poultry markets, including occupational exposure.

^d^Backyard poultry refers to persons exposed to household poultry raised in their backyard.

^e^The health outcomes for most cases were unknown because of no follow-up post hospitalization.

A total of 1189 environmental samples, comprising those from poultry drinking water or from cleaning poultry sewage, were collected from live poultry markets, poultry farms, poultry slaughterhouses, decentralized households, and wild bird habitats in Hubei province. Consequently, eight samples from environment (Table 2) were identified as positive for H7 subtype AIVs.

**Table 2:** H7N9 viruses sampled from the environment in Hubei province, China.

Isolate name	Collection date (YY-MM-DD)	Passage history	Collection area	
			City	Location
A/Environment/Hubei/12133/2017	2017-02-20	E1	Xiaogan	Poultry-raised backyard
A/Environment/Hubei/12134/2017	2017-02-20	E1	Xiaogan	Poultry-raised backyard
A/Environment/Hubei/12136/2017	2017-02-20	E2	Xiaogan	Poultry-raised backyard
A/Environment/Hubei/12137/2017	2017-02-18	E2	Jingmen	LPM^a^
A/Environment/Hubei/12167/2017	2017-02-16	E1	Enshi	LPM
A/Environment/Hubei/12176/2017	2017-02-16	E1	Ezhou	LPM
A/Environment/Hubei/12177/2017	2017-02-16	E1	Ezhou	LPM
A/Environment/Hubei/12178/2017	2017-02-16	E1	Ezhou	LPM

^a^LPM, live poultry market.

All samples were tested by real-time RT-PCR and inoculated into 9 to 11-day-old specific pathogen-free embryonated hens’ eggs in a biosafety level 3 laboratory at the Centers for Disease Control and Prevention (CDCs) where the influenza network laboratories distribute in Hubei province. All H7N9 sequences obtained in this study have been submitted to the GISAID database (platform.gisaid.org), with accession numbers listed in Table S1 in the Supplementary Material.

### Ethical approval

All experimental protocols including the hemagglutination inhibition (HI) assay and virus genome sequencing were performed according to the WHO and the China CDC protocols (detailed methods provided upon request), and were approved by the Laboratory Management Committee of the Hubei CDC. Hubei CDC is legally tasked with data collection on patients in the course of a public health investigation during an emerging infectious disease outbreak. Therefore, informed consent was waived.

### Phylogenetic analysis

All available HA (n = 1022 sequences; length = 1695 nt) and NA (n = 1018; 1398 nt) gene sequences of H7N9 (complete coding region) were downloaded from the GenBank (https://www.ncbi.nlm.nih.gov/genbank/) and GISAID databases (data collected on 27 March 2017). These sequences were aligned using MAFFT (v7.149)^9^. Maximum likelihood (ML) phylogenies of the HA and NA sequences were estimated using the GTR + I + Γ nucleotide substitution model in PhyML (v3.1)^10^. Node support was determined using the Shimodaira-Hasegawa (SH) approximate likelihood ratio test. All trees were rooted using the earliest sampled H7N9 avian influenza virus (A/Shanghai/1/2013) and visualized in FigTree (v1.4.3) (http://tree.bio.ed.ac.uk/software/figtree/).

## Results

### Characteristics of human H7N9 infections

In total, 14 human H7N9 cases in Hubei province (Tables 1 and 3) were studied, two of which were imported cases. All of the infected individuals had prior exposure to live poultry and experienced severe pneumonia and other symptoms commonly associated with H7N9 including fever, cough and shortness of breath. Ten of the infected individuals were exposed to live poultry markets (LPMs) or poultry transported from LPMs (two were poultry workers), while the remaining four cases were confirmed to have been exposed to backyard poultry.

**Table 3:** The epidemiological characteristics of 14 human cases of H7N9 virus infection in Hubei province.

Median age (Range), years		52 (26–78)
Gender, n (%)	Male	9 (64)
	Female	5 (36)
Occupation, n (%)	Farmer	3 (21)
	Retiree	3 (21)
	Poultry worker	2 (14)
	Other	6 (43)
Live poultry related exposure, n (%)	LPM^a^	10 (71)
	Backyard poultry	4 (29)

^a^LPM, live poultry market.

### Evolutionary relationships among H7N9 AIVs

**Figure 1:**
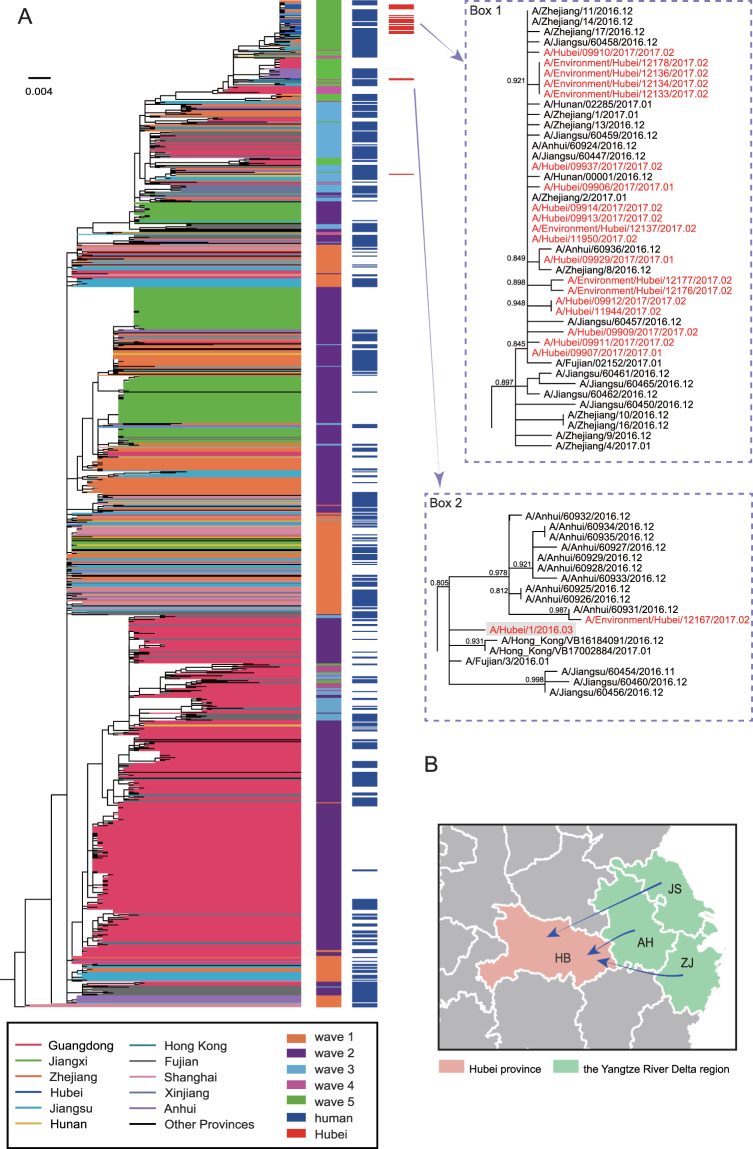
Phylogenetic tree of the HA gene of H7N9 influenza A viruses and their origins. (**A**) Viruses isolated from different Chinese provinces are distinguished by colors. The first column represents the five waves of H7N9 marked with different colors, while the second and last columns denote those viruses collected from humans and specifically from Hubei province. Those H7N9 viruses in Hubei (excluding strain A/Hubei/34007/2015 which was isolated in 2015) province are shown in detail in the dotted boxes and were colored in red. Box 1, the majority of isolates in Hubei province; box 2, the imported strain A/Hubei/1/2016 (highlighted) and its closely related environmental strain (A/Environment/Hubei/12167/2017) from 2017. The tree was rooted with virus A/Shanghai/1/2013. (**B**) A partial map indicating the routes of viral movement leading to the outbreak in Hubei province. Abbreviations are: HB, Hubei province; AH, Anhui province; JS, Jiangsu province; and ZJ, Zhejiang province. Blue arrow, Hubei strains mainly derived from Zhejiang, Jiangsu and Anhui provinces sampled in wave 5.

Tracing the origin of the H7N9 virus is of importance for effective prevention and surveillance strategies. We collected all available HA and NA gene sequences of H7N9 AIVs from GenBank and GISAID and performed a large-scale phylogenetic analysis. The HA (Figs 1; S1) and NA phylogenies (Fig. S2) revealed that the Hubei strains, including viruses isolated from both humans and the environment, generally clustered together and with H7N9 viruses from Zhejiang, Jiangsu and Anhui provinces sampled during wave 5, indicating that these viruses originated from the Yangtze River lineage.

### Imported H7N9 AIVs

Notably, the H7N9 virus A/Environment/Hubei/12167/2017 was phylogenetically distinct from the other Hubei strains, and clustered with human strains isolated in neighboring Anhui province, suggesting movement from Anhui to Hubei. In addition, it is striking that A/Environment/Hubei/12167/2017 was also related to human strain A/Hubei/1/2016 in both the HA and NA phylogenetic trees (Figs S1 and S2). A/Hubei/1/2016, sampled on March 2016 during, was isolated from a poultry worker in Nanjing city, Jiangsu province, who returned to Hubei and showed symptoms of influenza infection. Notably, the six internal genes of A/Hubei/1/2016 were closely related to A/Chicken/GZ79/2016 (H7N9) isolated from chickens in Ganzhou city, Jiangxi province (nucleotide identity, 99.56% for PB2, 99.25% for PB1, 99.95% for PA, 99.20% for NP, 99.59% for MP and 99.90% for NS). It therefore seems likely that an earlier H7N9 was imported into Hubei where it could establish itself in the local environment. Another clear example of importation into Hubei was A/Hubei/34007/2015, which clustered with H7N9 viruses from Anhui and Hunan provinces collected during wave 3 (Fig. 1; Figs S1 and S2). Interestingly, these viruses were isolated from a poultry seller who, in 2015, had sold birds imported from Anhui province into Hubei province.

### Amino acid substitutions in the Hubei H7N9 AIVs

We also investigated the molecular signatures of all the H7N9 AIVs isolated in Hubei province (Table 4). Mutations associated with resistance to oseltamivir or zanamivir, including E119V, I222L and R292K were not found in the NA proteins, such that all these viruses remain susceptible to these antivirals^8,11,12^. However, all viruses were resistant to amantadine because of the S31N mutation in M2 protein^8^. With respect to host-specificity, mutations T160A, G186V and Q226L, were observed in the HA protein of all 22 human and environmental samples, suggesting that the H7N9 viruses collected from Hubei province preferentially bind to human-type influenza receptors^8,11^. The majority of viruses isolated from humans acquired the E627K mutation in PB2 protein, which increases virulence in mice^8,11,13^. Similarity, the P42S mutation associated with virulence in H5N1^14^ was also identified in all viruses. Of note, the I368V mutation which would increase viral transmission in ferrets^15^ was present in the PB1 protein of all of the H7N9 viruses.

**Table 4:** Occurrence of amino acid substitutions associated with human adaptation of avian influenza viruses, increased virulence and antiviral drug resistance in the Hubei H7N9 strains.

Protein	Mutation	Amino acid (No. of strain)				Function
		Human samples		Environmental samples		
HA^a^	T160A	T(0)	A(14)	T(0)	A(8)	N-glycosylation loss and increased binding to human-type influenza receptor^11,13^
	G186V	G(0)	V(14)	G(0)	V(8)	Increased binding to human-type influenza receptor^8,13^
	Q226L	Q(0)	L(14)	Q(0)	L(8)	Increased binding to human-type influenza receptor^8,11,13^
M2^b^	S31N	S(0)	N(14)	S(0)	N(8)	Amantadine resistance^8,11,13^
PB2^b^	K526R	K(13)	R(1^c^)	K(7)	R(1^d^)	Enhances the function of 627K and 701N^8^
	E627K	E(5)	K(9^e^)			Enhanced polymerase activity and increased virulence in mice^8,11,13^
	D701N	D(12)	N(2^f^)	·		Nuclear Import^8,13^
PB1^b^	I368V	I(0)	V(14)	I(0)	V(8)	Increased transmission in ferrets^8,13^
PA^b^	V100A	V(12)	A(2^g^)	V(7)	A(1^h^)	Species-associated signature positions^8,13^
	K356R	K(0)	R(14)	K(0)	R(8)	Species-associated signature positions^8,13^
	S409N	S(0)	N(14)	S(0)	N(8)	Species-associated signature positions^8,13^
NS1^b^	P42S	P(0)	S(14)	P(0)	S(8)	Increased pathogenesis in mice^8,11,13^
	N205S	N(0)	S(14)	N(0)	S(8)	Altered antiviral response in host^8,13^

^a^According to the H3 numbering system.

^b^Internal genes were numbered from the start codon (M).

^c^Refers to human strain A/Hubei/1/2016.

^d^Refers to the environmental strain A/Environment/Hubei/12167/2017.

^e^Refers to the human strains. A/Hubei/09906/2017, A/Hubei/09907/2017, A/Hubei/09912/2017, A/Hubei/09913/2017, A/Hubei/09914/2017, A/Hubei/09929/2017, A/Hubei/09937/2017, A/Hubei/11944/2017 and A/Hubei/11950/2017.

^f^Refers to the human strains A/Hubei/1/2016 and A/Hubei/34007/2015.

^g^Refers to human strains A/Hubei/1/2016 and A/Hubei/34007/2015.

^h^Refers to the environmental strains A/Environment/Hubei/12167/2017.

## Discussion

Hubei province, central China, did not experience *in situ* transmission during the first four waves of infection by H7N9 avian influenza virus. However, human H7N9 cases began to be reported in Hubei in January 2017, including several fatalities. This abrupt emergence of H7N9 influenza during wave 5 has attracted considerable attention. Our study documented that the sudden increase of human cases of H7N9 in Hubei in 2017 was due to one single importation event originating from the Yangtze River lineage. Notably, however, that one environmental sample from 2017 contains a virus that is closely related to a human virus from 2016 (March) suggests that the environmental viral gene pool may be complex, and that H7N9 viruses could have circulated in the local environment prior to the 2017outbreak. Finally, it is striking that all the human cases had exposure to live poultry, indicating that control measures with a focus on hygienic management of LPMs and cross-regional transportation should be strengthened.

## Electronic supplementary material

Supplementary materials

Dataset 1
