# Publisher Correction: Sudden emergence of human infections with H7N9 avian influenza A virus in Hubei province, central China

**DOI:** 10.1038/s41598-018-25257-3

**Published:** 2018-05-10

**Authors:** Jiafa Liu, Junqiang Xu, Linlin Liu, Xiaoman Wei, Yi Song, Bin Fang, Xiao Yu, Xiang Li, Guojun Ye, Yingying Du, Mingyue Chen, Weifeng Shi, Di Liu, Edward C. Holmes, Jie Cui

**Affiliations:** 1 Hubei Provincial Center for Disease Control and Prevention, Wuhan, 430079 China; 2 CAS Key Laboratory of Special Pathogens and Biosafety, Center for Emerging Infectious Diseases, Wuhan Institute of Virology, Chinese Academy of Sciences, Wuhan, 430071 China; 3 University of Chinese Academy of Sciences, Beijing, 100049 China; 4 Institute of Pathogen Biology, Taishan Medical College, Taian, Shandong 271000 China; 5 Center for Influenza Research and Early-Warning (CASCIRE), Chinese Academy of Sciences, Beijing, 100101 China; 6 CAS Key Laboratory of Pathogenic Microbiology and Immunology, Institute of Microbiology, Chinese Academy of Sciences, Beijing, 100101 China; 7 Savid Medical School, University of Chinese Academy of Sciences, Beijing, 101408 China; 8 Marie Bashir Institute for Infectious Diseases and Biosecurity, Charles Perkins Centre, School of Life and Environmental Sciences and Sydney Medical School, The University of Sydney, Sydney, New South Wales Australia

Correction to: *Scientific Reports* 10.1038/s41598-018-20988-9, published online 06 February 2018

In the original PDF version of this Article, Figure 1B was incorrectly displayed. This has now been corrected in the PDF; the HTML version of the paper was correct from the time of publication.

