# Supplementary material for: Sudden emergence of human infections with H7N9 avian influenza A virus in Hubei province, central China
Source: Sci Rep. 2018 Feb 6;8:2486. doi: 10.1038/s41598-018-20988-9 (PMC5802767; doi:10.1038/s41598-018-20988-9)

Sudden emergence of human infections with H7N9 avian influenza A virus in Hubei province, central China

Jiafa Liu[1#], Junqiang Xu[1#], Linlin Liu[1#], Xiaoman Wei[2,3#], Yi Song[1], Bin Fang[1], Xiao Yu[1], Xiang Li[1], Guojun Ye[1], Yingying Du[2], Mingyue Chen[2], Weifeng Shi[4], Di Liu[5,6,7], Edward C. Holmes[8], Jie Cui[2]*

[1]Hubei Provincial Center for Disease Control and Prevention, Wuhan 430079, China.

[2]CAS Key Laboratory of Special Pathogens and Biosafety, Center for Emerging Infectious Diseases, Wuhan Institute of Virology, Chinese Academy of Sciences, Wuhan 430071, China.

[3]University of Chinese Academy of Sciences, Beijing 100049, China.

[4]Institute of Pathogen Biology, Taishan Medical College, Taian, Shandong 271000, China.

[5]Center for Influenza Research and Early-Warning (CASCIRE), Chinese Academy of Sciences, Beijing 100101, China.

[6]CAS Key Laboratory of Pathogenic Microbiology and Immunology, Institute of Microbiology, Chinese Academy of Sciences, Beijing 100101, China.

[7]Savid Medical School, University of Chinese Academy of Sciences, Beijing 101408, China.

[8]Marie Bashir Institute for Infectious Diseases and Biosecurity, Charles Perkins Centre, School of Life and Environmental Sciences and Sydney Medical School, The

University of Sydney, Sydney, New South Wales, Australia.

[#]These authors contributed equally to this manuscript.

*Correspondence: Jie Cui (jiecui@wh.iov.cn)

Supplementary materials

Figure S1. Phylogenetic tree of H7N9 avian influenza virus using HA gene sequences. A total of 1022 H7N9 viruses collected from 2013-2017 in China were analyzed. The tree is rooted to A/Shanghai/1/2013(H7N9). The scale bar represents the number of nucleotide substitutions per site (subs/site). The H7N9 viruses collected from Hubei province are marked in red and the remaining H7N9 viruses are marked in black.

Figure S2. Phylogenetic tree of H7N9 avian influenza virus using NA gene sequences. A total 1018 viruses of different H7N9 collected from 2013-2017 in China were analyzed. The tree is rooted to A/Shanghai/1/2013(H7N9). Bootstrap values lower than 70% are not shown. The scale bar represents the number of nucleotide substitutions per site (subs/site). The H7N9 viruses collected from Hubei province marked in red and the remaining H7N9 viruses are marked in black.

Supplementary Tables

Additional data table S1 (separate file)

Accession numbers of all sequences of H7N9 viruses in Hubei province.

Figure S1

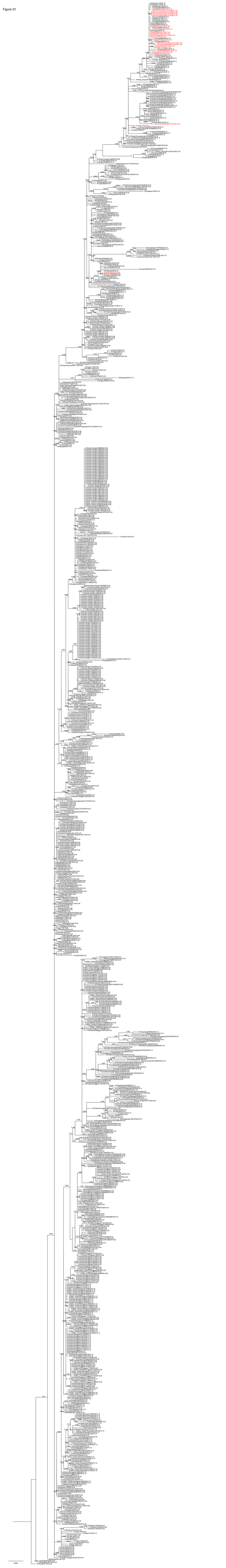

Figure S2

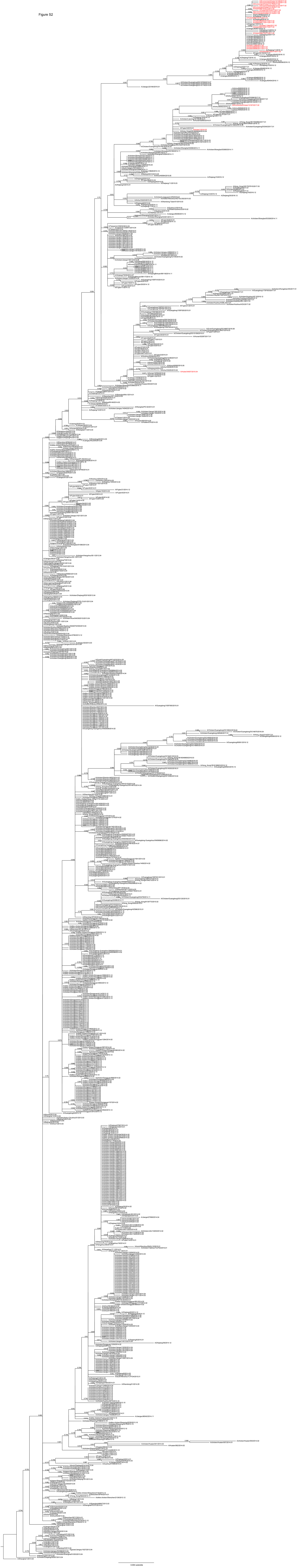

Supplement: Supplementary file 1 — Supplementary materials [file 41598_2018_20988_MOESM1_ESM.pdf]
